# Aldehyde *N*,*N*-dimethylhydrazone-based fluorescent substrate for peroxidase-mediated assays†

**DOI:** 10.1039/d2ra00087c

**Published:** 2022-03-21

**Authors:** Soyeon Yoo, Sudeok Kim, Sangyeon Jeon, Min Su Han

**Affiliations:** Department of Chemistry, Gwangju Institute of Science and Technology (GIST) Gwangju 61005 Republic of Korea happyhan@gist.ac.kr

## Abstract

Numerous assays based on peroxidase activity have been developed for the detection of analytes due to the various optical peroxidase substrates. However, most substrates are sensitive to light and pH and are over-oxidized in the presence of excess H_2_O_2_. In this study, 2-((6-methoxynaphthalen-2-yl)methylene)-1,1-dimethylhydrazine (MNDH), a fluorescent peroxidase substrate prepared from naphthalene-based aldehyde *N*,*N*-dimethylhydrazone, was developed. MNDH showed quantitative fluorescence changes with respect to the H_2_O_2_ concentration in the presence of horseradish peroxidase (HRP), and the MNDH/HRP assay showed no changes in fluorescence caused by over-oxidation in the presence of excess H_2_O_2_. Further, MNDH was thermo- and photostable. Additionally, the assay could be operated over a considerably wide pH range, from acidic to neutral. Moreover, MNDH can be used to detect glucose quantitatively in human serum samples by using an enzyme cascade assay system.

## Abstract 2

A fluorescent peroxidase substrate is devised based on aldehyde *N*,*N*-dimethylhydrazone with no over-oxidation by excess H_2_O_2_, high photo stability, and wide operating pH.

## Graphical abstract

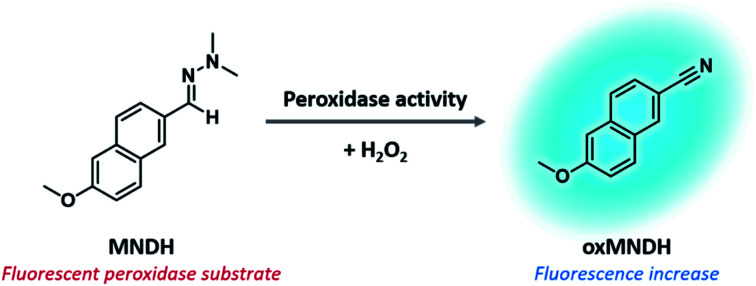

## Introduction

Peroxidase activity, which involves the oxidation of organic substrates with the aid of H_2_O_2_, is extensively utilized in various bio-sensors, such as multi-enzyme cascade-based assays and enzyme-linked immunosorbent assays.^1–3^ The widespread application of peroxidases in sensing systems can be attributed to (1) the large number of optical peroxidase substrates, (2) the very high turnover numbers of these catalyst systems, and (3) the ease of combining peroxidases with other enzymes that produce H_2_O_2_ as a byproduct.^4^ Horseradish peroxidase (HRP), a natural heme-containing enzyme, is one of the most commonly used enzymes in sensing systems owing to its high specificity, sensitivity, and stability for conjugation with antibodies.^4–6^ HRP is oxidized to oxHRP by using hydrogen peroxide, which then catalyzes the oxidation of the optical substrate, while oxHRP is reduced to HRP.^7^ The resulting changes in the optical properties of the substrate enable qualitative and quantitative analyses of the analyte.

As optical substrates undergo changes in color, fluorescence, or chemiluminescence in the presence of peroxidases, these systems have been widely applied in sensing systems. Commonly used peroxidase substrates include colorimetric substrates such as 2,2′-azino-bis(3-ethylbenzothiazoline-6-sulfonate) (ABTS) and 3,3,5,5-tetramethylbenzidine (TMB), fluorescent substrates such as Amplex Red, and chemiluminescent substrates such as luminol.^8–11^ Thus, a wide range of substrates having different optical properties exists, and suitable substrates can be selected in accordance with assay operating conditions such as pH. Colorimetric substrates are mainly used in acidic-to-neutral conditions, and the color changes are detectable by the naked eye.^12–14^ However, in complex matrices such as biological samples, the colored radical products of ABTS and TMB formed through peroxidase-mediated oxidation can react with other antioxidants, resulting in a loss of color.^15,16^ In contrast, fluorescent and chemiluminescent substrates have higher sensitivities, and thus, lower concentrations can be used.^17–19^ However, fluorescent substrates such as Amplex Red can only be used at pH 7–8 because oxidized Amplex Red, a fluorescent resorufin, is further oxidized to the non-fluorescent resazurin under acidic conditions.^20^ The chemiluminescence of luminol is also inhibited at an acidic pH.^21^ In addition, in the case of Amplex Red, if a small amount of its product (resorufin) is present, photo-oxidation occurs even in the absence of H_2_O_2_, thereby lowering the detection sensitivity.^22^ Moreover, these common substrates are excessively oxidized by high concentrations of H_2_O_2_, resulting in a loss of color or fluorescence.^23,24^ Hence, new fluorescent peroxidase substrates must be (1) universally applicable at an acidic-to-neutral pH, (2) photostable, and (3) not overly oxidized by excess H_2_O_2_.

**Scheme 1:**
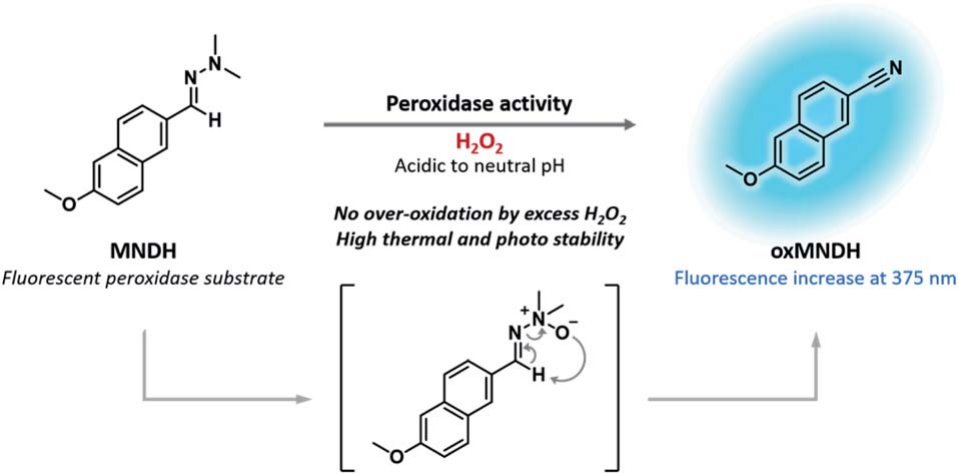
Structure of MNDH, a new fluorescent peroxidase substrate, and the proposed mechanism of the peroxidase-catalyzed oxidation of MNDH in the presence of H_2_O_2_.

In this study, 2-((6-methoxynaphthalen-2-yl)methylene)-1,1-dimethylhydrazine (MNDH) was developed as a new fluorescent peroxidase substrate that can be used under acidic-to-neutral pH conditions. MNDH was designed by combining the reactive aldehyde *N*,*N*-dimethylhydrazone moiety and the fluorophore methoxynaphthalene (Scheme 1). *N*,*N*-Dimethylhydrazone is a well-known protecting group for ketones but is not used to protect aldehydes because complete deprotection is difficult.^25^ The aldehyde *N*,*N*-dimethylhydrazone moiety can be oxidized by employing H_2_O_2_ in the presence of methyltrioxorhenium (MTO) as a catalyst to form an *N*-oxide intermediate, which is converted to a nitrile through the Cope elimination reaction.^26,27^ This reaction was performed under mild conditions using a small amount of acetic acid. Because HRP mainly oxidizes the amine group of common substrates with the help of H_2_O_2_, we speculated that, instead of MTO, HRP could be used to catalyze the oxidation of *N*,*N*-dimethylhydrazone under mild conditions at an acidic pH. In that case, the nitrile group formed by the oxidation reaction would not be overly oxidized by the remaining H_2_O_2_, thus solving the over-oxidation problem.^26^ As expected, MNDH was oxidized by HRP and H_2_O_2_ to form oxMNDH with a nitrile group, resulting in an increase in the fluorescence intensity at 375 nm. Based on this change in fluorescence, we found that MNDH can be used to measure the peroxidase activity of HRP at pH 4 and 7, and we confirmed that there was no change in the nonspecific fluorescence signal arising from the presence of excessive H_2_O_2_ or light and heat.

## Experimental

### Materials and instrumentation

Chemical reagents were purchased from commercial sources (Sigma-Aldrich, Tokyo Chemical Industry, and Duksan Pure Chemical, Korea) and used without further purification. Human serum was purchased from Sigma-Aldrich. ^1^H and ^13^C nuclear magnetic resonance (NMR) spectra were recorded using a JEOL 400 MHz NMR spectrometer. High-resolution mass spectra (HRMS) were recorded using a Bruker Impact II quadruple time-of-flight (QToF) mass spectrometer with an electrospray ionization (ESI) source. Melting point analysis was performed using a Büchi M-560 melting point apparatus. The fluorescence spectra were recorded using an Agilent Cary Eclipse fluorescence spectrophotometer, and absorbance spectra were recorded on a JASCO V-630 UV-Vis spectrophotometer. Fourier-transform infrared (FTIR) spectra were recorded on a Thermo Scientific NICOLET iS10 spectrometer using a KBr disc (Thermo Scientific, 25 × 4 mm).

### Synthesis of 2-((6-methoxynaphthalen-2-yl)methylene)-1,1-dimethylhydrazine (MNDH)

6-Methoxy-2-naphthaldehyde (10 mmol, 1.86 g) was dissolved in 30 mL of dichloromethane containing anhydrous magnesium sulfate (20 mmol, 2.41 g), *N*,*N*-dimethylhydrazine hydrochloride (20 mmol, 1.45 g), and trimethylamine (7 mmol, 0.98 mL). The mixture was then stirred for 10 h at room temperature. Subsequently, the mixture was washed three times with a saturated NaHCO_3_ (aq.) solution and brine. Then, the organic layer was dried using sodium sulfate and concentrated under reduced pressure. The mixture was then purified by applying flash column chromatography (CHCl_3_ : MeOH = 100 : 1), followed by two-solvent recrystallization (ethyl acetate/hexane) to yield a white crystalline solid (1.62 g, yield 70.96%). ^1^H-NMR (400 MHz, DMSO-*D*_6_) *δ* 7.81–7.72 (m, 4H), 7.43 (s, 1H), 7.28 (d, *J* = 2.5 Hz, 1H), 7.13 (dd, *J* = 8.9, 2.5 Hz, 1H), 3.86 (s, 3H), 2.93 (s, 6H). ^1^H-NMR (400 MHz, acetonitrile (ACN)-*D*_3_) *δ* 7.82 (dd, *J* = 8.5, 1.5 Hz, 1H), 7.77 (s, 1H), 7.73 (dd, *J* = 12.4, 8.7 Hz, 2H), 7.43 (s, 1H), 7.24 (d, *J* = 2.4 Hz, 1H), 7.13 (dd, *J* = 9.0, 2.6 Hz, 1H), 3.92–3.87 (3H), 2.97–2.93 (6H). ^13^C-NMR (101 MHz, DMSO-*D*_6_) *δ* 157.2, 133.7, 132.5, 132.4, 129.2, 128.5, 126.9, 124.8, 123.3, 118.7, 106.2, 55.2, 42.6. HRMS (ESI): [M + H]^+^*m*/*z* calculated for C_14_H_17_N_2_O^+^: 229.1335; found: 229.1333. Melting point: 144.1–144.8 °C.

### pH screening of the MNDH/HRP system

MNDH (20 μM, ACN 5%) was added to various buffer solutions (20 mM) having different pH ranges (4.0–5.0 (acetate), 6.0 (MES), and 7.0–9.0 (Tris–HCl)), containing HRP (50 mU mL^−1^) and H_2_O_2_ (0 or 50 μM). Fluorescence spectra were recorded over 1 h at 2 min intervals at 25 °C.

### Study of the operating mechanism of the MNDH/HRP system

Combinations of MNDH (20 μM, ACN 5%), HRP (50 mU mL^−1^), and H_2_O_2_ (50 μM) were added to an acetate buffer solution (20 mM) of pH 4.0, and the fluorescence spectra of samples were recorded at 25 °C for 5 min.

For large-scale sample preparation, MNDH (100 μM, ACN 5%), HRP (250 mU mL^−1^), and H_2_O_2_ (250 μM) in a pH 4.0 buffer solution (acetate, 20 mM) were placed in a 500 mL volumetric flask. The mixture was extracted three times by using chloroform. The organic layer was then collected and concentrated under reduced pressure, and the oxMNDH was obtained by performing column chromatography using chloroform. ^1^H-NMR (400 MHz, ACN-*D*_3_) *δ* 8.27 (s, 1H), 7.89 (t, *J* = 8.9 Hz, 2H), 7.62 (dd, *J* = 8.5, 1.8 Hz, 1H), 7.35 (d, *J* = 2.4 Hz, 1H), 7.28 (dd, *J* = 8.9, 2.4 Hz, 1H), 3.94 (s, 3H). ^13^C-NMR (101 MHz, ACN-*D*_3_) *δ* 161.1, 137.5, 134.8, 131.0, 128.9, 128.6, 128.0, 121.5, 120.4, 107.4, 107.1, 56.3. The FTIR spectra of MNDH and oxMNDH were obtained by using the KBr disc method.

### MNDH and H_2_O_2_ titration using the MNDH/HRP system

MNDH (0 to 30 μM, ACN 5%) was added to a buffer solution (acetate pH 4.0 or Tris–HCl pH 7.0, 20 mM) containing HRP (50 mU mL^−1^) and H_2_O_2_ (50 μM), and the fluorescence spectra were recorded at 25 °C at 15 s intervals over 10 min for the pH 4.0 and 60 min for the pH 7.0.

H_2_O_2_ (0–30 or 20 μM) was added to buffer solution (acetate pH 4.0 or Tris–HCl pH 7.0, 20 mM) containing HRP (50 mU mL^−1^) and MNDH (20 μM, ACN 5%), and fluorescence spectra were recorded at 25 °C at 15 s intervals over 10 min for the pH 4.0 and 60 min for the pH 7.0.

The initial reaction velocity (*V*_0_) was calculated from the initial linear change in the fluorescence intensity from 0 to 60 s at pH 4.0 and from 0 to 5 min at pH 7.0. The Michaelis–Menten constant (*K*_m_) and maximum reaction velocity (*V*_max_) were obtained from the Lineweaver–Burk plot.

### Thermo- and photostability of MNDH

A batch of MNDH solutions (400 μM, ACN 100%) was incubated for 6 h at 60 °C on a hot plate. Another batch of MNDH solutions (400 μM, ACN 100%) was incubated for 24 h in a light-box with a 25 W light-emitting diode lamp (6000 K). Then, the MNDH (20 μM, ACN 5%) incubated on the hot plate or in a light-box was added to the buffer solution (acetate pH 4.0 or Tris–HCl pH 7.0, 20 mM) containing HRP (50 mU mL^−1^) and H_2_O_2_ (50 μM), and fluorescence spectra were recorded at 25 °C for 5 min for the pH 4.0 and 30 min for the pH 7.0.

### Application of the MNDH/HRP system for glucose detection

Glucose (0–100 μM) was added to a buffer solution (Tris–HCl pH 7.0, 20 mM) containing HRP (0.1 U mL^−1^), glucose oxidase (GOx) (0.5 U mL^−1^), and MNDH (20 μM, ACN 5%), and fluorescence spectra were recorded at 25 °C for 60 min at 30 s intervals.

### Selectivity for glucose over other saccharides

Various saccharides (50 μM glucose and 500 μM each of galactose (Gal), fructose (Fru), maltose (Mal), lactose (Lac), and sucrose (Suc)) were added to a buffer solution (Tris–HCl pH 7.0, 20 mM) containing HRP (0.1 U mL^−1^), GOx (0.5 U mL^−1^), and MNDH (20 μM, ACN 5%), and fluorescence spectra were recorded at 25 °C for 60 min at 30 s intervals.

### Serum glucose assay using the MNDH/HRP system

Human serum samples were prepared by applying ultra-filtration and diluted by a factor of 37.5. The samples were then added to the buffer solution (Tris–HCl pH 7.0, 20 mM) containing HRP (0.1 U mL^−1^), GOx (0.5 U mL^−1^), and MNDH (20 μM, ACN 5%), and fluorescence spectra were recorded at 25 °C for 30 min at 30 s intervals.

## Results and discussion

### Synthesis and characterization of MNDH as the peroxidase substrate

MNDH was synthesized in a single step, yielding a white crystalline solid, and the ^1^H- and ^13^C-NMR spectra, HRMS, and melting point data (Fig. S1–S3†) confirmed that MNDH had been cleanly synthesized. After synthesis, we investigated the pH range over which MNDH can be used as a peroxidase substrate *via* reaction with H_2_O_2_. Under various pH conditions (pH 4.0 to 9.0), time-dependent changes in the fluorescence of MNDH solutions containing HRP were observed in both the presence and absence of H_2_O_2_ (Fig. S4†). In the absence of H_2_O_2_, no change was observed in the fluorescence of the solution over time, regardless of the pH. In the presence of H_2_O_2_, the use of a lower pH resulted in a rapid increase in the fluorescence intensity of the solution over time. In particular, the fluorescence signal was saturated within 3 min at pH 4.0. Although the change in the absolute fluorescence intensity of the solution at pH 7.0 was slower and lower than that at pH 4.0, the difference in the fluorescence intensity in the presence of H_2_O_2_ was approximately 65-times greater after 5 min than that in its absence (at pH 7.0). This is because the fluorescence intensity at 375 nm for MNDH was almost zero at pH 7.0. Consequently, MNDH can be used as a fluorescent substrate in acidic-to-neutral solutions.

### Mechanistic study of MNDH

**Fig. 1:**
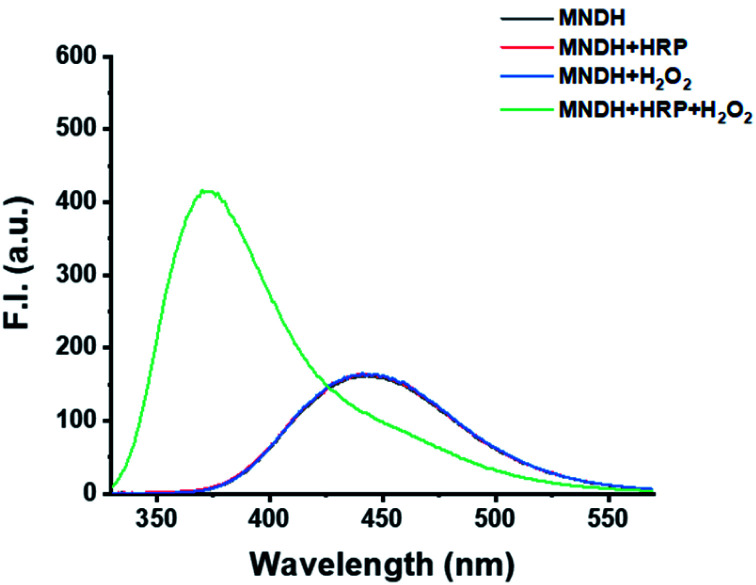
Fluorescence spectra of MNDH in buffer solution containing various combinations of HRP and H_2_O_2_. [MNDH] = 20 μM, [H_2_O_2_] = 50 μM, [HRP] = 50 mU mL^−1^, [acetate pH 4.0] = 20 mM. F.I. = fluorescence intensity.

To determine the mechanism of the MNDH/HRP system, the changes in the fluorescence of MNDH in a pH 4.0 buffer solution containing a combination of HRP and H_2_O_2_ were observed. The fluorescence spectra of the MNDH solutions containing either HRP or H_2_O_2_ were consistent with those of the MNDH solutions without both HRP and H_2_O_2_ (Fig. 1). In contrast, for the MNDH solution containing both HRP and H_2_O_2_, the fluorescence intensity at 375 nm increased significantly. Thus, the fluorescence of MNDH was affected only in the presence of both HRP and H_2_O_2_. This indicates that oxHRP, which is formed in the presence of H_2_O_2_, catalyzes the oxidation of MNDH, suggesting that MNDH can be used as a new fluorescent substrate for peroxidases. To confirm the structure of the oxidized form of MNDH (oxMNDH) formed in the presence of H_2_O_2_ and HRP, a large-scale sample was prepared, and ^1^H- and ^13^C-NMR and FTIR measurements were performed. As shown in the ^1^H-NMR spectra (Fig. S2 and S5†), the peaks corresponding to the dimethyl and N=CH moieties of the dimethylhydrazone group of MNDH disappear after oxidation to yield oxMNDH. In addition, in the ^13^C-NMR spectra (Fig. S6†), no peaks corresponding to the dimethyl group are observed. Furthermore, the obtained NMR spectra of oxMNDH are found to be consistent with the NMR spectra of 6-methoxy-2-naphthonitrile (Fig. S7 and S8†),^27,28^ and the IR spectrum of oxMNDH contains a new peak at 2222 cm^−1^, which can be attributed to a C≡N group (Fig. S9†). These results reveal that oxMNDH has the structure of 6-methoxy-2-naphthonitrile (C_12_H_9_NO). Therefore, we conclude that the *N*,*N*-dimethylhydrazone moiety in MNDH was oxidized to a nitrile group in the presence of HRP and H_2_O_2_*via* the Cope elimination reaction per our original design.

### Kinetic study of the enzymatic reaction and H_2_O_2_ detection in the MNDH/HRP system

**Fig. 2:**
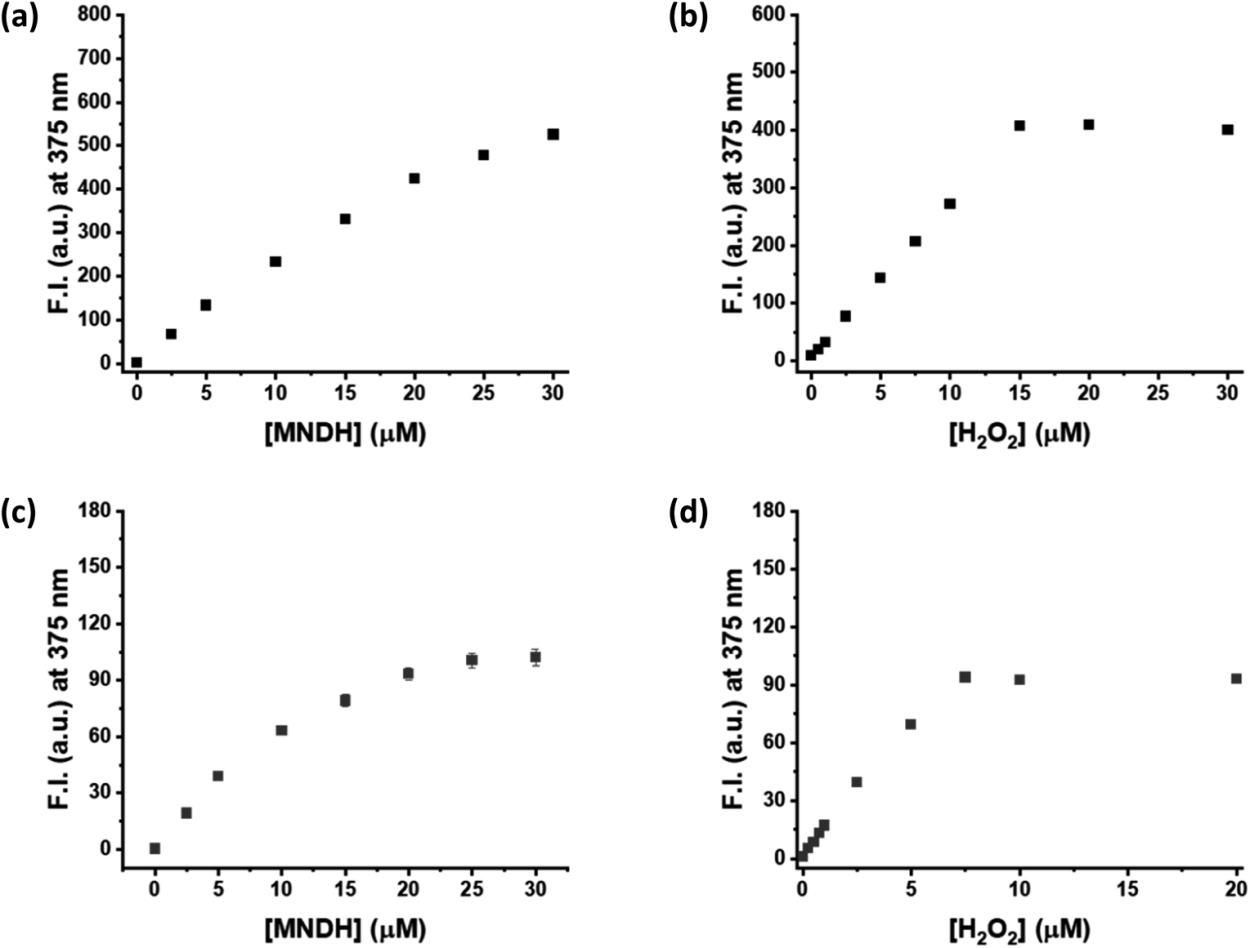
Fluorescence intensity (F.I.) *versus* concentration for assays of (a and c) MNDH or (b and d) H_2_O_2_ at a fixed concentration of the other in the presence of HRP at (a and b) pH 4.0 or (c and d) pH 7.0. [MNDH] = 20 μM, [H_2_O_2_] = 50 μM, [HRP] = 50 mU mL^−1^, [acetate pH 4.0, Tris–HCl pH 7.0] = 20 mM.

Next, a kinetic study of the peroxidase reaction was conducted using MNDH as the peroxidase substrate. The samples were prepared by fixing the concentration of either MNDH or H_2_O_2_ while changing the concentration of the other; these experiments were performed in a pH 4.0 or 7.0 buffer solution containing HRP. Concentration-dependent fluorescence of the assay was observed for both MNDH and H_2_O_2_. In particular, as the concentration of MNDH or H_2_O_2_ increased, the fluorescence intensity of the solution and the initial rate (*V*_0_) of the enzymatic reaction increased (Fig. 2, S10 and S11†). At pH 4.0, MNDH was rapidly oxidized by HRP and H_2_O_2_ to the extent that the fluorescence intensity was saturated within approximately 3 min, and the fluorescence increased significantly when the MNDH concentration was 20–30 μM (Fig. 2a and S10†). In addition, for H_2_O_2_ concentrations of 0–15 μM, the concentration-dependent change in the fluorescence intensity was linear, and the limit of detection (LOD) reached as low as 0.03 μM (Fig. 2b and S12a†). At pH 7.0, the fluorescence intensity increased relatively slowly with time (Fig. S11†). The increase was linear between H_2_O_2_ concentrations of 0 and 7.5 μM, and the LOD was 0.06 μM (Fig. 2b and S12b†). As shown by these LOD values, low concentrations of H_2_O_2_ can be detected by the MNDH/HRP system. Next, we used the Michaelis–Menten equation to obtain the kinetic parameters. When the reciprocal of *V*_0_ was plotted against the reciprocal of MNDH or H_2_O_2_ concentration, linear plots having excellent correlation coefficients (*R*^2^; 0.956–0.996) were obtained. As shown in Table 1, *K*_m_ and *V*_max_ are 18.14 μM and 0.38 ΔF s^−1^, respectively, for H_2_O_2_, and 0.66 μM and 7.11 ΔF s^−1^, respectively, for MNDH, at pH 4.0. These *K*_m_ values are much lower than those of other widely used colorimetric substrates (for example, 0.434 mM for TMB and 3.70 mM for H_2_O_2_) but similar to those for Amplex Red.^29,30^ Unfortunately, the *K*_m_ value for MNDH is rather high at pH 7.0, although that for H_2_O_2_ is low: 313.19 μM. Moreover, in the presence of excess H_2_O_2_, the saturated fluorescence signal of oxMNDH remains high for a long period, suggesting that oxMNDH is not over-oxidized by the remaining H_2_O_2_ (Fig. S10a and b†). Hence, these results indicate that MNDH is a promising new fluorescent peroxidase substrate.

**Table 1:** Comparison of the kinetic parameters of HRP depending on MNDH and H_2_O_2_ concentrations, including Michaelis–Menten constants (*K*_m_) and maximum reaction rates (*V*_max_) at pH 4.0 and 7.0

Enzyme	pH	Substrate	*K*_m_ (μM)	*V*_max_ (ΔF s^−1^)
HRP	4.0	H_2_O_2_	18.14	0.38
		MNDH	0.66	7.11
	7.0	H_2_O_2_	313.19	0.08
		MNDH	952.70	0.20

### Thermo- and photostability of MNDH

**Fig. 3:**
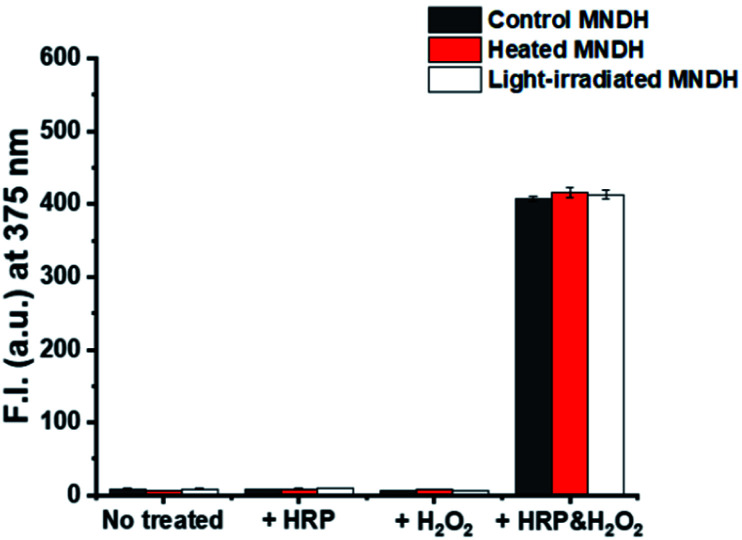
Fluorescence intensity (F.I.) of MNDH previously exposed to heat and light in buffer solutions containing various combinations of HRP and H_2_O_2_. [MNDH] = 20 μM, [H_2_O_2_] = 50 μM, [HRP] = 50 mU mL^−1^, [acetate pH 4.0] = 20 mM.

The stability of the peroxidase substrate in the presence of light and heat during storage and use is crucial because, on receiving energy from these sources, unstable substrates can undergo a range of reactions that can diminish their fluorescence properties. These problems reduce the accuracy and precision of analyte detection, thereby affecting the reliability of the assay. For example, Amplex Red, which is a widely used substrate, has low photostability and requires protection from light during use, making it an inconvenient reagent. To verify the thermostability of our system, the MNDH solution was heated on a hot plate at 60 °C for 6 h and then cooled to room temperature. The change in the fluorescence of the MNDH solution after adding it to a combination of H_2_O_2_ and HRP in a pH 4.0 buffer solution was then measured at 25 °C (Fig. 3). The fluorescence signal of the sample containing preheated MNDH was similar to that of unheated MNDH, indicating thermostability. Next, the photostability of MNDH was tested. After exposure to 25 W, 6000 K light for 24 h in a light-box, the fluorescence of the MNDH solution was measured using the same method as that used for the thermostability experiments; no changes were observed, indicating that the MNDH solution is photostable. Further, in the absence of HRP and H_2_O_2_, the light-irradiated MNDH showed no changes in fluorescence, indicating that the MNDH had not been photo-oxidized. These results were also observed at pH 7.0 (Fig. S13†). Although MNDH had a lower emission wavelength than previously reported fluorescent peroxidase substrates such as Amplex Red, it exhibited comparatively higher thermo- and photostability.^31–34^ Therefore, as a result of its easier storage, MNDH can be used as a substitute for Amplex Red.

### Application of MNDH for glucose detection

**Fig. 4:**
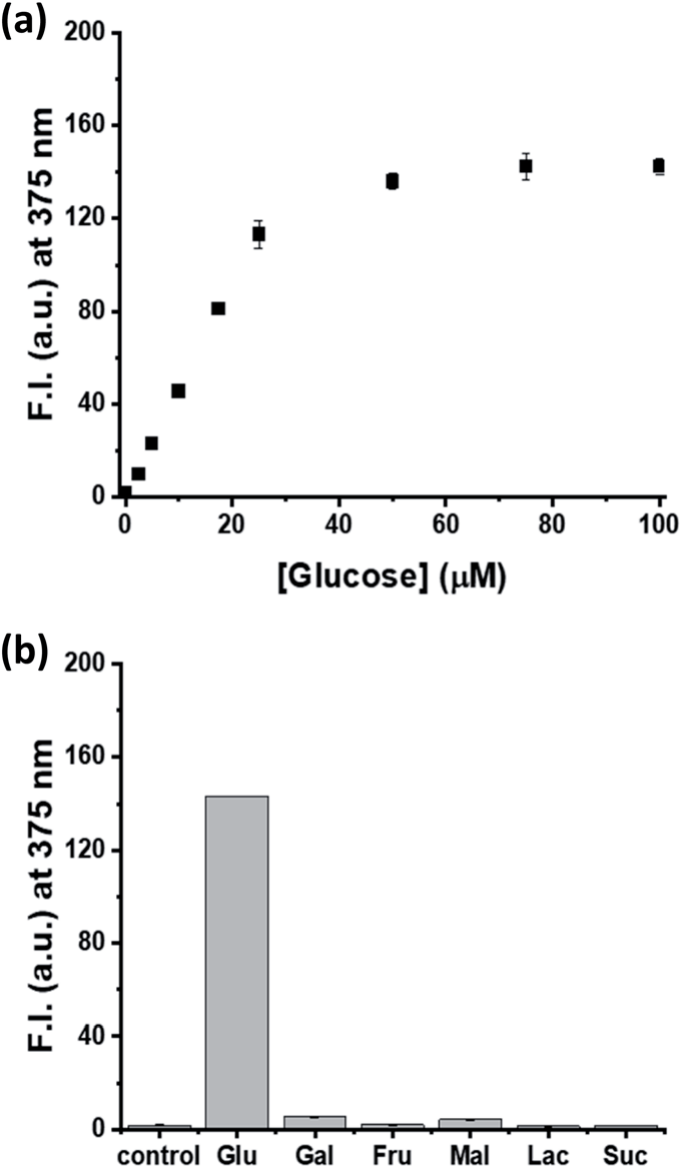
Fluorescence intensity (F.I.) of assay solutions *versus* (a) concentration of glucose or (b) type of saccharide. [MNDH] = 20 μM, [glucose] = 50 μM, [other saccharide] = 500 μM, [HRP] = 100 mU mL^−1^, [GOx] = 500 mU mL^−1^, [Tris–HCl pH 7.0] = 20 mM.

Finally, a model study based on glucose detection was conducted to confirm if MNDH can be applied as a peroxidase substrate for multi-enzyme cascade assays. After glucose was added at various concentrations to a pH 7.0 buffer solution containing GOx, HRP, and MNDH, the fluorescence intensity was measured. As the glucose concentration increased, the fluorescence intensity increased and, finally, became saturated (Fig. 4a and S14a†). The plot of fluorescence intensity *versus* glucose concentration showed excellent linearity (*R*^2^ = 0.987) between glucose concentrations of 0 and 20 μM, having a low LOD of 0.31 μM (Fig. S15†). In addition, the selectivity of the MNDH/GOx/HRP assay for glucose in the presence of other saccharides, including Gal, Fru, Mal, Lac, and Suc, was tested. The concentrations of the other saccharides were maintained at 10 times the concentration of glucose. After the samples were prepared in the same manner as the glucose titration, the fluorescence intensities of the samples were measured. As shown in Fig. 4b and S14b,† unlike glucose, the other saccharides do not cause any significant increase in fluorescence, indicating that the MNDH/GOx/HRP assay system is highly glucose-specific. The applicability of the MNDH/GOx/HRP assay system to human serum samples was also tested. Human serum samples were purchased from Sigma-Aldrich and prepared by applying ultrafiltration to remove proteins. The MNDH/GOx/HRP assay system was used to determine the concentration of glucose in human serum to be 5.04 ± 0.01 mM, which was nearly identical to that (5.19 ± 0.03 mM) measured by using a glucose meter. Thus, the proposed system can accurately measure the concentration of glucose in human serum, indicating its potential for application in the diagnosis of diseases for which glucose is a biomarker.

## Conclusions

MNDH, a new fluorescent peroxidase substrate based on the aldehyde *N*,*N*-dimethylhydrazone, was developed. The synthesis of MNDH is simple in a single step and requires commercially available reagents. MNDH was oxidized to fluorescent oxMNDH containing a cyano group by using HRP and H_2_O_2_, and the MNDH/HRP assay system can be operated at an acidic-to-neutral pH. Further, the detection of H_2_O_2_ at micromolar levels is possible. MNDH showed considerable thermo- and photostability and was not over-oxidized in the presence of excess H_2_O_2_. Moreover, MNDH can be applied to an enzyme cascade assay system for the detection of glucose in human serum. Thus, MNDH is expected to replace existing peroxidase substrates in various sensing fields.

## Author contributions

Soyeon Yoo: data curation, formal analysis, investigation, methodology, validation, visualization, writing – original draft, writing – review & editing. Sudeok Kim: data curation, investigation. Sangyeon Jeon: methodology, resources. Min Su Han: conceptualization, funding acquisition, project administration, supervision, writing – review & editing.

## Conflicts of interest

There are no conflicts to declare.

## Supplementary Material

RA-012-D2RA00087C-s001
