# Supplementary material for: Aldehyde N,N-dimethylhydrazone-based fluorescent substrate for peroxidase-mediated assays
Source: RSC Adv. 2022 Mar 21;12(14):8668–73. doi: 10.1039/d2ra00087c (PMC8984872; doi:10.1039/d2ra00087c)
Supplement: RA-012-D2RA00087C-s001 [file RA-012-D2RA00087C-s001.pdf]

Electronic Supplementary Material (ESI) for RSC Advances.
This journal is © The Royal Society of Chemistry 2022

Supplementary information

Aldehyde N,N-dimethylhydrazone-based fluorescent substrate for peroxidase-mediated assays

Soyeon Yoo, Sudeok Kim, Sangyeon Jeon and Min Su Han*

Department of Chemistry, Gwangju Institute of Science and Technology (GIST), Gwangju 61005, Republic of Korea

* Corresponding author. E-mail: happyhan@gist.ac.kr; Tel.: +82 62 715 2848; Fax: +82 62 715 2866

Supplementary results

NMR spectra

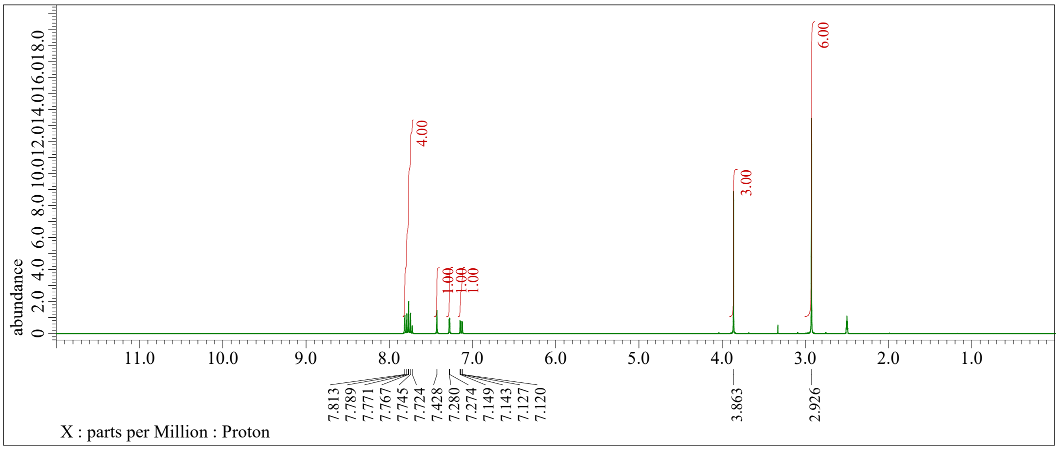

Fig. S1 ^{1}H NMR (400 MHz, DMSO-d6) of **MNDH**.

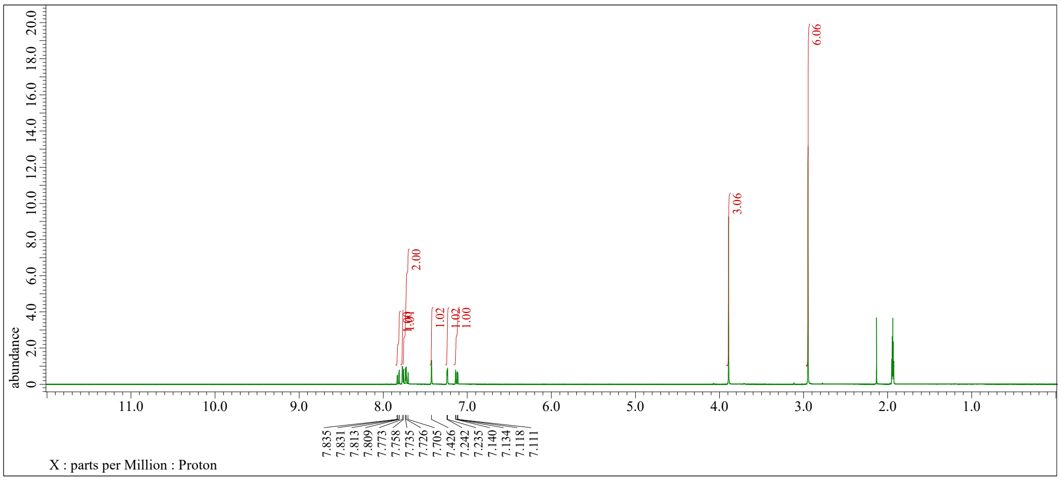

Fig. S2 ^{1}H NMR (400 MHz, ACN-d3) of **MNDH**.

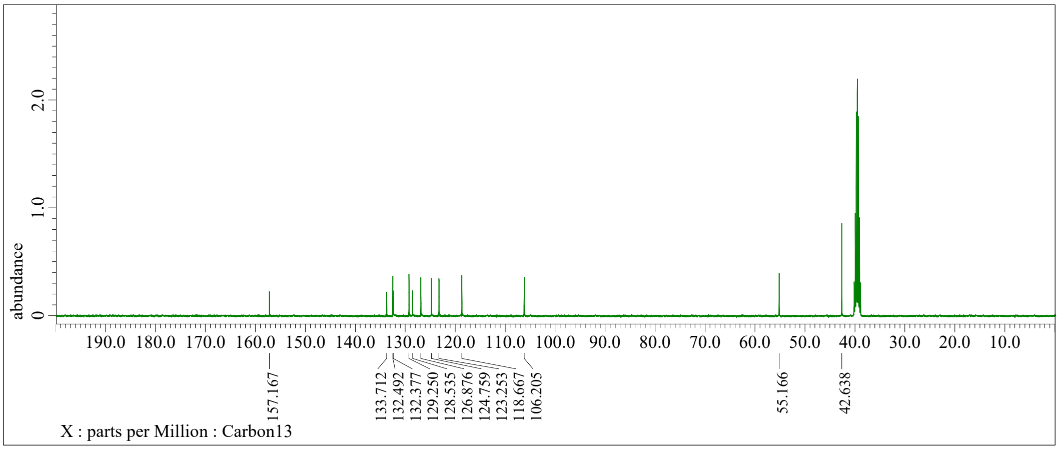

Fig. S3 ^{13}C NMR (101 MHz, DMSO-d6) of **MNDH**.

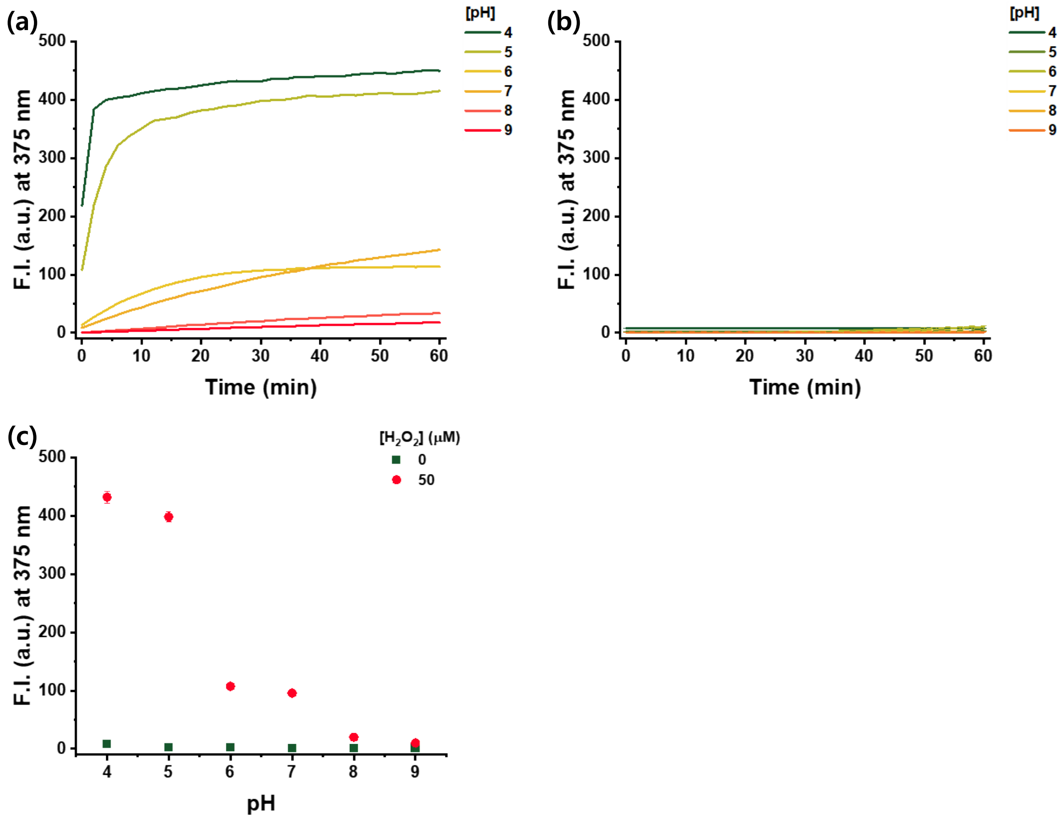

Fig. S4 Fluorescence intensity versus time for **MNDH** in the various pH buffer solution containing the HRP and (a) with or (b) without H_2O_2. (c) Plot of fluorescence intensity for assay solutions versus pH in the present or absent of H_2O_2. [**MNDH**] = 20 μM, [H_2O_2] = 50 μM, [HRP] = 50 mU/mL, [Acetate pH 4-5, MES pH 6 and Tris-HCl pH 7-9] = 20 mM.

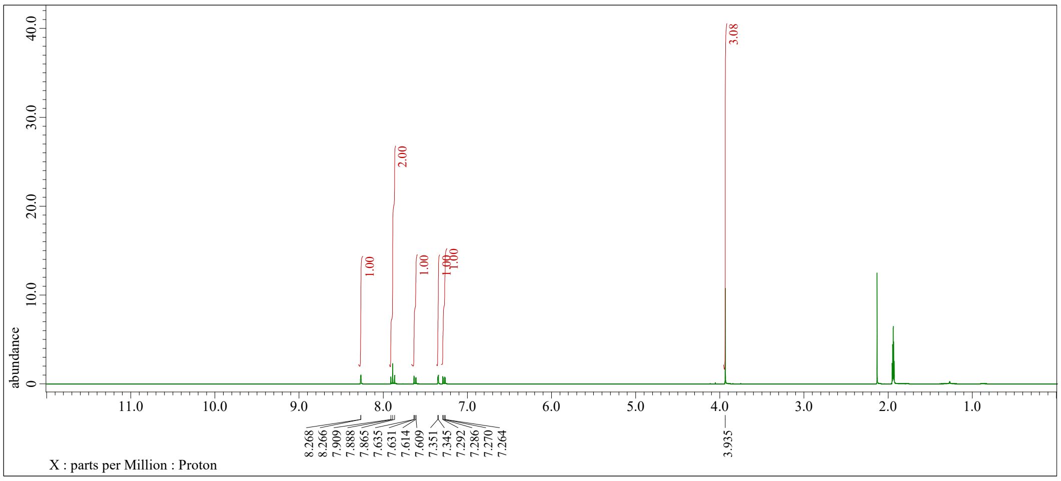

Fig. S5 ^{1}H NMR (400 MHz, ACN-d3) of **oxMNDH**.

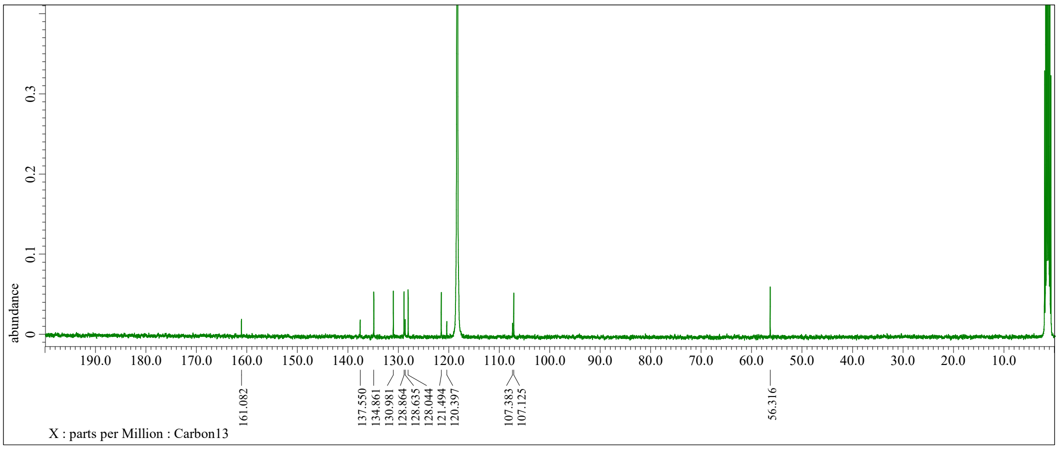

Fig. S6 ^{13}C NMR (101 MHz, ACN-d3) of ox**MNDH**.

Synthesis of 6-Methoxy-2-naphthonitrile

A 6-Methoxy-2-naphthonitrile was synthesized following the previous literature.[1] The methyltrioxorhenium (0.004 mmol, 0.001 g) and H_2O_2 (1.85 mmol, 0.16 mL) were added to the ethanol (1 mL) and cooled at – 30 °C. After MNDH (0.87 mmol, 0.20 g) dissolved in dichloromethane (1 mL) was added dropwise to above the mixture, the mixture was stirred at – 30 °C for 2 hr. The product was extracted with ethyl acetate. The organic layer was dried using sodium sulfate and concentrated under reduced pressure. The mixture was then purified by column chromatography ($CHCl_3$) to yield a white crystalline solid. ^{1}H-NMR (400 MHz, ACN-d3) δ 8.26 (d, J = 0.9 Hz, 1H), 7.88 (t, J = 9.0 Hz, 2H), 7.62 (dd, J = 8.5, 1.8 Hz, 1H), 7.34 (d, J = 2.4 Hz, 1H), 7.27 (dd, J = 9.0, 2.6 Hz, 1H), 3.93 (s, 3H). ^{13}C-NMR (101 MHz, ACN-d3) δ 161.0, 137.5, 134.8, 131.0, 128.8, 128.6, 128.0, 121.4, 120.4, 107.3, 107.1, 56.3.

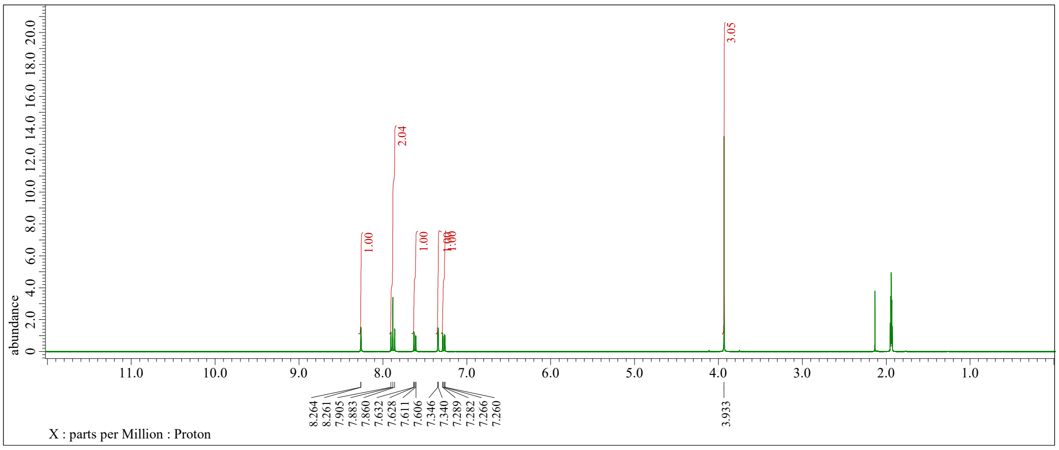

Fig. S7 ^{1}H NMR (400 MHz, ACN-d3) of 6-methoxy-2-naphthonitrile.

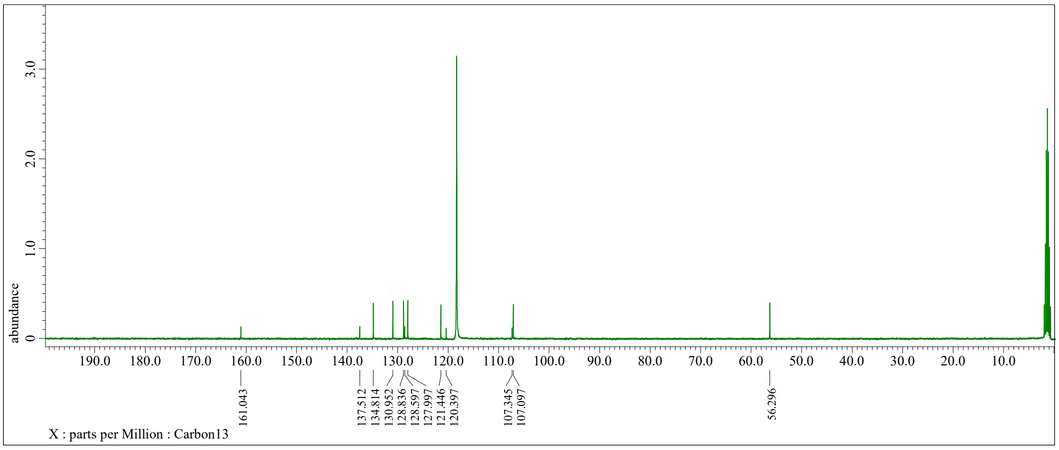

Fig. S8 ^{13}C NMR (400 MHz, ACN-d3) of 6-methoxy-2-naphthonitrile.

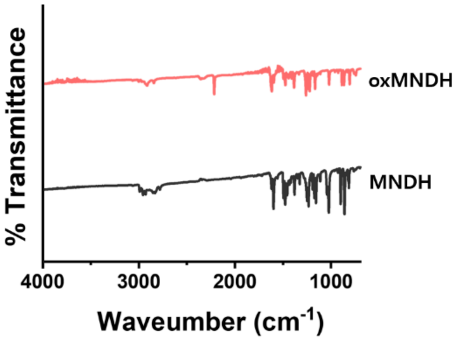

Fig. S9 FT-IR spectra of **MNDH** and ox**MNDH**.

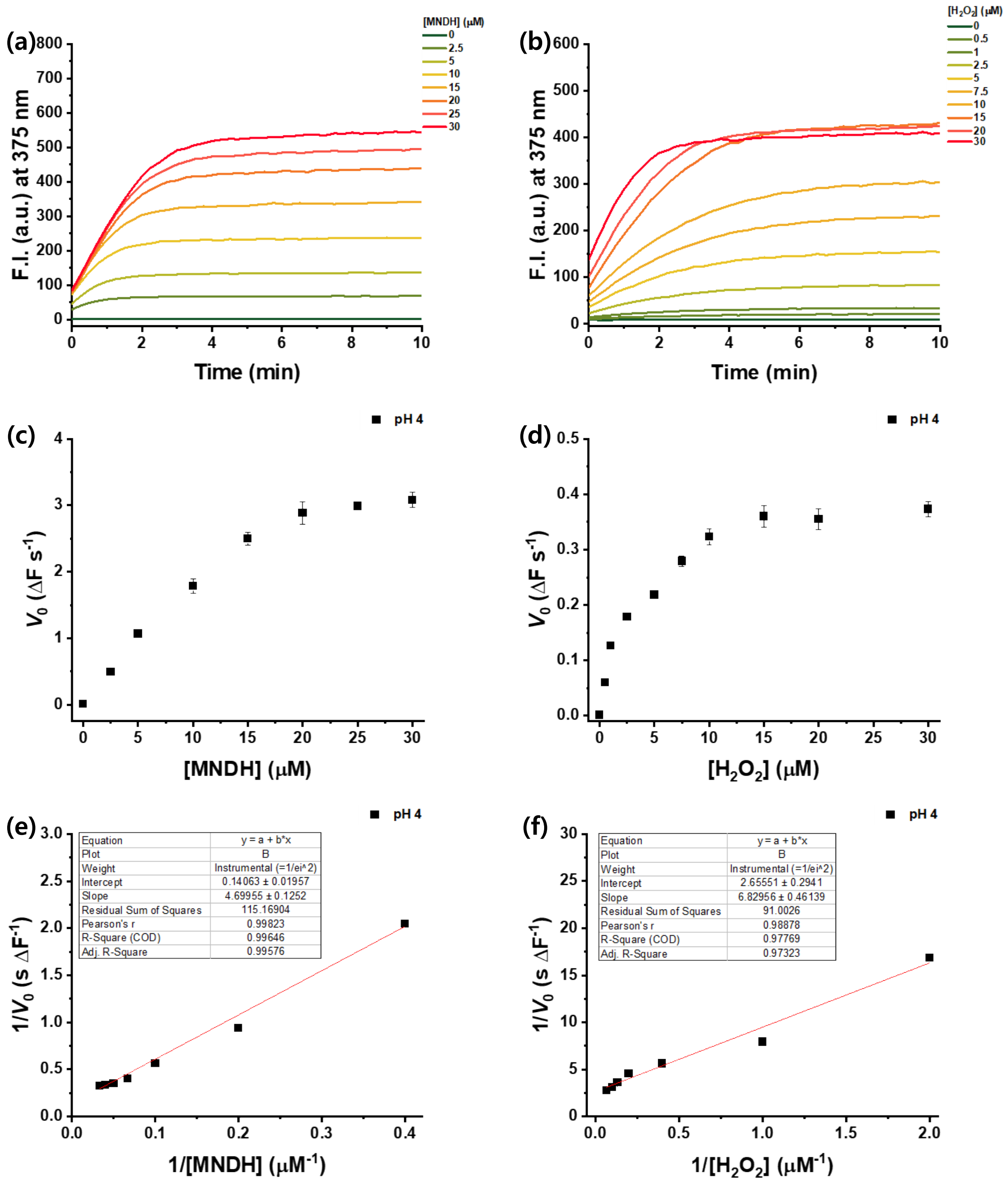

Fig. S10 Fluorescence intensity versus time for the assay solution containing the various concentration of (a) **MNDH** or (b) H_2O_2 at a fixed concentration of the other, in the presence of HRP, at pH 4. Michaelis-Menten plots of the assay solution versus the various concentrations of (c) **MNDH** or (d) H_2O_2. Double reciprocal plots of the peroxidase-like activity of assay solution versus concentration of (e) **MNDH** or (f) H_2O_2. [**MNDH**] = 20 μM, [H_2O_2] = 50 μM, [HRP] = 50 mU/mL, [Acetate pH 4.0] = 20 mM.

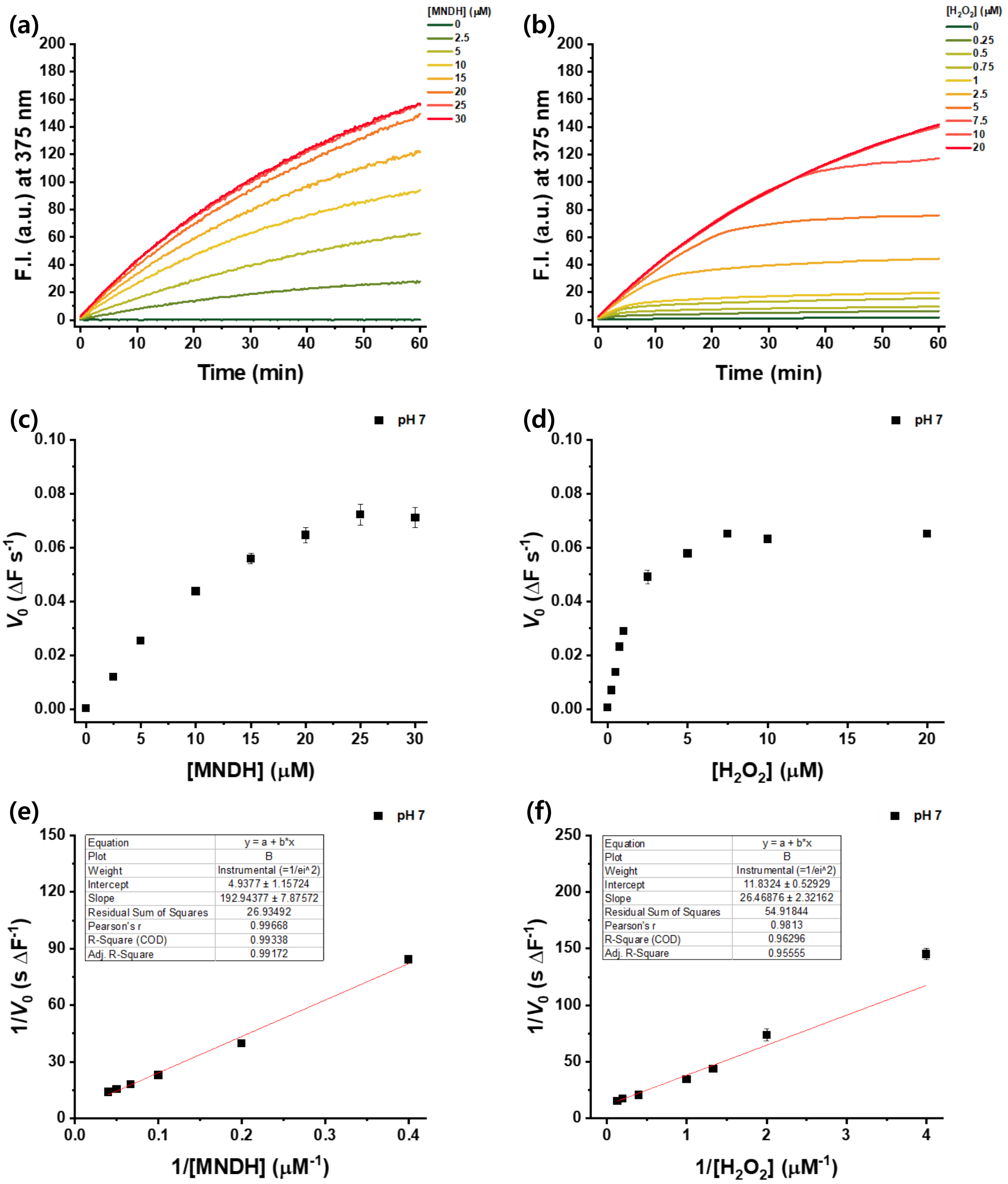

Fig. S11 Fluorescence intensity versus time for the assay solution containing the various concentration of (a) **MNDH** or (b) H_2O_2 at a fixed concentration of the other, in the presence of HRP, at pH 7. Michaelis-Menten plots of the assay solution versus the various concentrations of (c) **MNDH** or (d) H_2O_2. Double reciprocal plots of the peroxidase-like activity of assay solution versus concentration of (e) **MNDH** or (f) H_2O_2. [**MNDH**] = 20 μM, [H_2O_2] = 50 μM, [HRP] = 50 mU/mL, [Tris-HCl pH 7.0] = 20 mM.

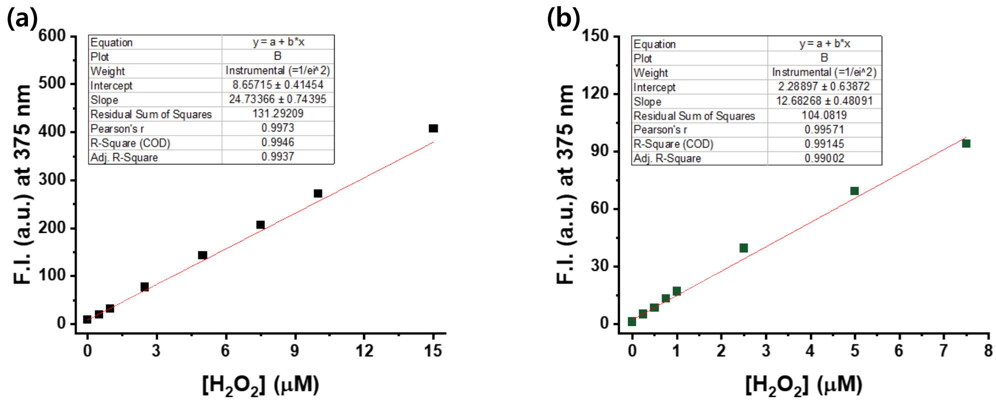

Fig. S12 Linear range of H_2O_2 titration at (a) pH 4 and (b) pH 7. [**MNDH**] = 20 μM, [H_2O_2] = 50 μM, [HRP] = 50 mU/mL, [Acetate pH 4.0 and Tris-HCl pH 7.0] = 20 mM.

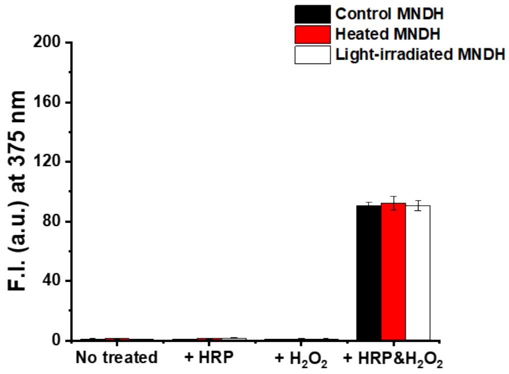

Fig. S13 Plot of fluorescence intensity of **MNDH** previously exposed to heat and light in buffer solutions containing various combinations of HRP and H_2O_2. [**MNDH**] = 20 μM, [H_2O_2] = 50 μM, [HRP] = 50 mU/mL, [Tris-HCl pH 7.0] = 20 mM.

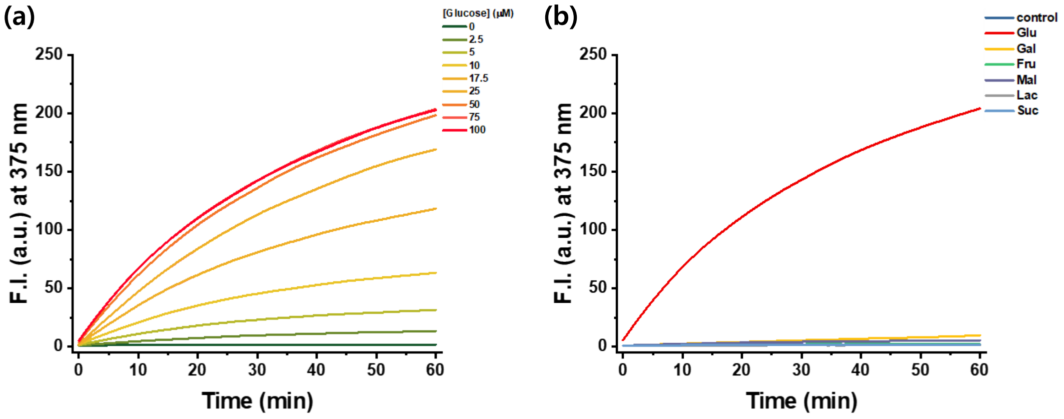

Fig. S14 Fluorescence intensity versus time for assay solutions versus (a) concentration of glucose or (b) type of saccharide. [**MNDH**] = 20 μM, [glucose] = 50 μM, [other saccharide] = 500 μM, [HRP] = 100 mU/mL, [GOx] = 500 mU/mL, [Tris-HCl pH 7.0] = 20 mM.

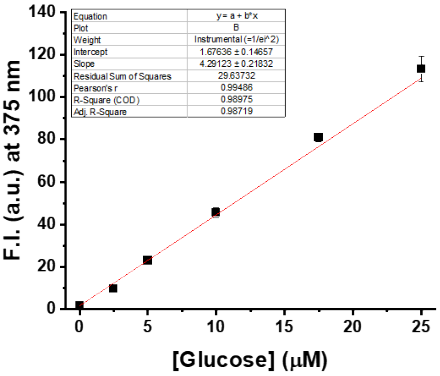

Fig. S15 Linear range of glucose titration. [**MNDH**] = 20 μM, [HRP] = 100 mU/mL, [GOx] = 500 mU/mL, [Tris-HCl pH 7.0] = 20 mM.

Reference

1. H. Rudler, B. Denise and S. Masi, *C. R. Acad. Sci. Paris, Serie IIc, Chimie/Chemistry*, 2000, **3**, 793-801.
